# Clarifying the Role of the Rostral dmPFC/dACC in Fear/Anxiety: Learning, Appraisal or Expression?

**DOI:** 10.1371/journal.pone.0050120

**Published:** 2012-11-26

**Authors:** Simon Maier, Anna Szalkowski, Susanne Kamphausen, Evgeniy Perlov, Bernd Feige, Jens Blechert, Alexandra Philipsen, Ludger Tebartz van Elst, Raffael Kalisch, Oliver Tüscher

**Affiliations:** 1 Department of Psychiatry and Psychotherapy, University Medical Center Freiburg, Freiburg im Breisgau, Germany; 2 Institute of Biology III, Faculty of Biology, University of Freiburg, Freiburg, Germany; 3 Department of Psychiatry and Psychotherapy, University Medical Centre Tuebingen, Tuebingen, Germany; 4 Department of Clinical Psychology, Psychotherapy and Health Psychology, Institute of Psychology, University of Salzburg, Salzburg, Austria; 5 Institute for Systems Neuroscience, University Medical Center Hamburg-Eppendorf (UKE), Hamburg, Germany; 6 Department of Psychiatry and Psychotherapy, University Medical Centre Mainz, Mainz, Germany; University of Missouri-Kansas City, United States of America

## Abstract

Recent studies have begun to carve out a specific role for the rostral part of the dorsal medial prefrontal cortex (dmPFC) and adjacent dorsal anterior cingulate cortex (dACC) in fear/anxiety. Within a novel general framework of dorsal mPFC/ACC areas subserving the appraisal of threat and concomitant expression of fear responses and ventral mPFC/ACC areas subserving fear regulation, the rostral dmPFC/dACC has been proposed to specifically mediate the conscious, negative appraisal of threat situations including, as an extreme variant, catastrophizing. An alternative explanation that has not been conclusively ruled out yet is that the area is involved in fear learning. We tested two different fear expression paradigms in separate fMRI studies (study 1: instructed fear, study 2: testing of Pavlovian conditioned fear) with independent groups of healthy adult subjects. In both paradigms the absence of reinforcement precluded conditioning. We demonstrate significant BOLD activation of an identical rostral dmPFC/dACC area. In the Pavlovian paradigm (study 2), the area only activated robustly once prior conditioning had finished. Thus, our data argue against a role of the area in fear learning. We further replicate a repeated observation of a dissociation between peripheral-physiological fear responding and rostral dmPFC/dACC activation, strongly suggesting the area does not directly generate fear responses but rather contributes to appraisal processes. Although we succeeded in preventing extinction of conditioned responding in either paradigm, the data do not allow us to definitively exclude an involvement of the area in fear extinction learning. We discuss the broader implications of this finding for our understanding of mPFC/ACC function in fear and in negative emotion more generally.

## Introduction

The mPFC/ACC is among the areas most consistently activated by emotional stimuli [1], [2] and lesioning or stimulating the mPFC/ACC has a profound impact on emotional behavior [3]. An early, popular idea that dorsal mPFC/ACC areas are involved in “cold” cognitive processing and control of emotion while ventral areas (vmPFC and adjacent subgenual ACC) process “hot” affective information and generate emotional responses [4] has recently been challenged [5]–[7]. Etkin et al. [5] proposed a new functional segregation of the mPFC/ACC specifically for negative emotions such as fear/anxiety (defined as the emotional reaction observed during anticipation or expectation of a potential harmful event) in which dorsal areas evaluate (appraise) emotional information and generate appropriate responses whereas ventral areas are involved in response regulation. A further conjecture is that the mPFC/ACC is not critically involved in simple Pavlovian forms of learning, in particular in the acquisition of fear conditioning and extinction.

The present series of studies served to elucidate the contribution to fear/anxiety processing of a rostral sub-region of the dmPFC/dACC that is located approximately at the level of the genu of the corpus callosum or more anterior and whose importance has been highlighted by recent work on instructed fear (IF) [8]–[10]. In IF paradigms, subjects are told before the experiment that a given conditioned stimulus (CS) will or may be followed by an unconditioned stimulus (UCS). Hence, learning takes place before the experiment and fear responding to the CS is a result of the conscious appraisal of the CS as threatening, on the basis of explicit CS-UCS contingency knowledge [11]. IF paradigms consistently activate the dorsal (but not the ventral) mPFC/ACC, with typical IF activations including both rostral dmPFC/dACC and a relatively more posterior part extending into the presupplemental motor area (preSMA) (see [10] for a meta-analysis). The rostral dmPFC/dACC activations fall into an area that only responds to threat when subjects have enough time to think about the threatening situation [9] and is hyperactive in subjects that over-perceive or over-interpret their own threat reactions in a catastrophizing-like manner [12]. In both studies, activation changes in this area were not paralleled by changes in heart rate or skin conductance measures of fear, suggesting the area is not directly involved in the generation, or expression, of fear responses but specifically in conscious threat appraisal. The more posterior IF activations, by contrast, are also found in uninstructed fear (UF), that is, during Pavlovian conditioning [10], where they correlate with conditioned skin conductance responses (SCRs) [13], [14]. Lesion and electrical stimulation studies further round up the picture of mid-to-posterior dmPFC/dACC as a generator of physiological arousal responses, including in fear [15]–[17]. More globally speaking, these data support the appraisal/expression theory of dorsal mPFC/ACC function proposed by Etkin et al. [5] but they do not exclude that the dorsal mPFC/ACC might also contribute to fear learning, a possibility highlighted by evidence that the dACC is involved in action-outcome learning (e.g. [18]).

The IF paradigm previously used by us involved occasional CS-UCS pairings that served to maintain the credibility of the instruction across repeated CS trials [8], [9], [19], [20]. We were thus unable to rule out that additional reinforcement learning (i.e., Pavlovian conditioning) took place during testing and that this might explain the observed dmPFC/dACC activations. In the first of two fMRI studies (study 1), we therefore abstained from presenting any UCS during IF testing. We asked whether this would still evoke activation in our rostral dmPFC/dACC region of interest (ROI), which would further substantiate a role for this area in fear appraisal and/or expression. In fMRI study 2, we conducted classical Pavlovian conditioning followed by testing of the ensuing UF (i.e., of conditioned responding) in the absence of further reinforcement by the UCS. The test phase thus again allowed us to investigate fear appraisal/expression unconfounded by fear learning and to ask whether this evokes rostral dmPFC/dACC activation. Moreover, by analyzing rostral dmPFC/dACC activation time courses across acquisition and testing in study 2 we were able to examine a potential additional contribution of this area to fear learning.

**Figure 1:**
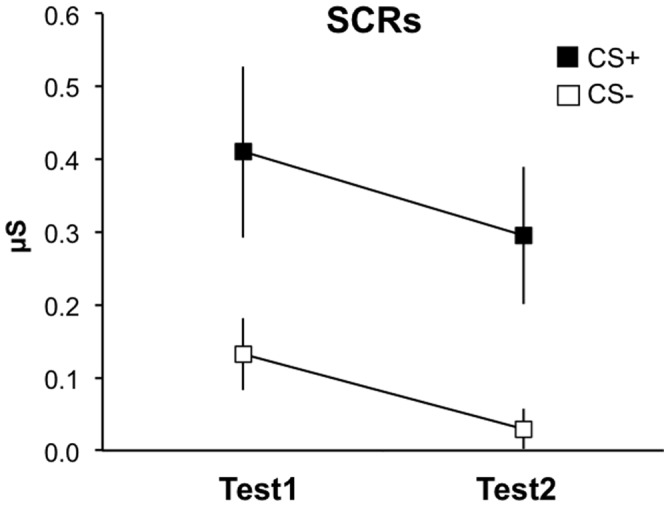
Instructed fear (study 1): Skin conductance. SCRs show stable threat responding across the two test runs (IF-Test1, IF-Test2). Error bars: s.e.m.

## Materials and Methods

### Subjects

We report data from an IF and a separate UF study that were primarily conducted for other purposes (fear expression in ADHD patients compared to normal healthy controls, under review) than dealt with in this paper and therefore used partly different stimuli, procedures and scanning protocols. We emphasize that this precludes any formal comparison of the two data sets. For study 1 (IF), 22 healthy normal subjects were recruited of which five could not be analyzed due to technical problems (MRI artifacts, excessive head movement or faulty skin conductance recordings). The remaining 17 subjects (11 female) had an average age of 35 years (range 23 to 46). 3 subjects had finished a general secondary school, 7 an intermediate secondary school and 7 a grammar school. Average intelligence measured by the Mehrfachwahl-Wortschatz-Intelligenz-Test (MWT-B [21]) was 111.9±19.4. For study 2 (UF), a separate cohort of 24 subjects was recruited of which seven could not be analyzed due to technical problems. The remaining 17 subjects (10 female) had an average age of 31 years (range 23 to 57) and an average intelligence of 116.6±14.4. 4 participants had attended an intermediate secondary school and 13 a grammar school. None showed any axis I or II diagnoses, as assessed by a trained psychologist using the Structured Clinical Interview for DSM-IV [22], [23].

**Figure 2:**
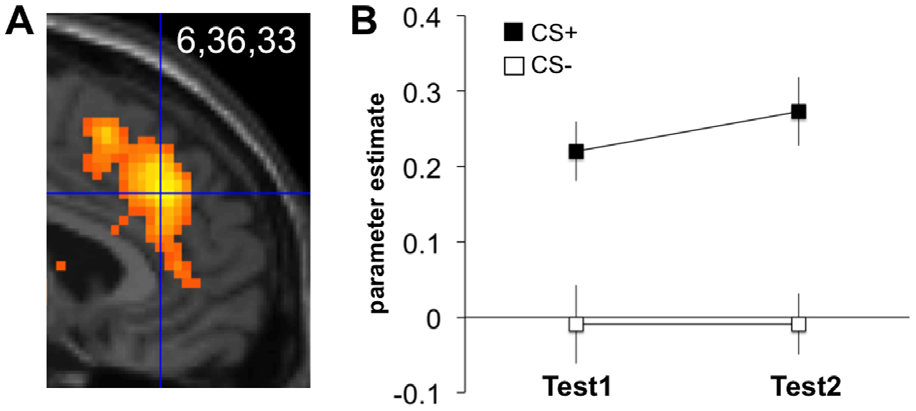
Instructed fear (study 1): Rostral dmPFC/dACC activation. (**A**) Contrast ‘CS+>CS−’ across both test runs (IF-Test1, IF-Test2) (model 1, see **Methods**). Display threshold: p<0.001 uncorrected. Activations superimposed on a canonical structural image. (**B**) Parameter estimates from the peak voxel, estimated separately for each test run (model 2). Error bars: s.e.m.

### Ethics Statement

All subjects gave written informed consent prior to participation, and the study was approved by the ethics committee of the University Medical Center Freiburg (Approval ID: EK-Freiburg 60/07).

**Figure 3:**
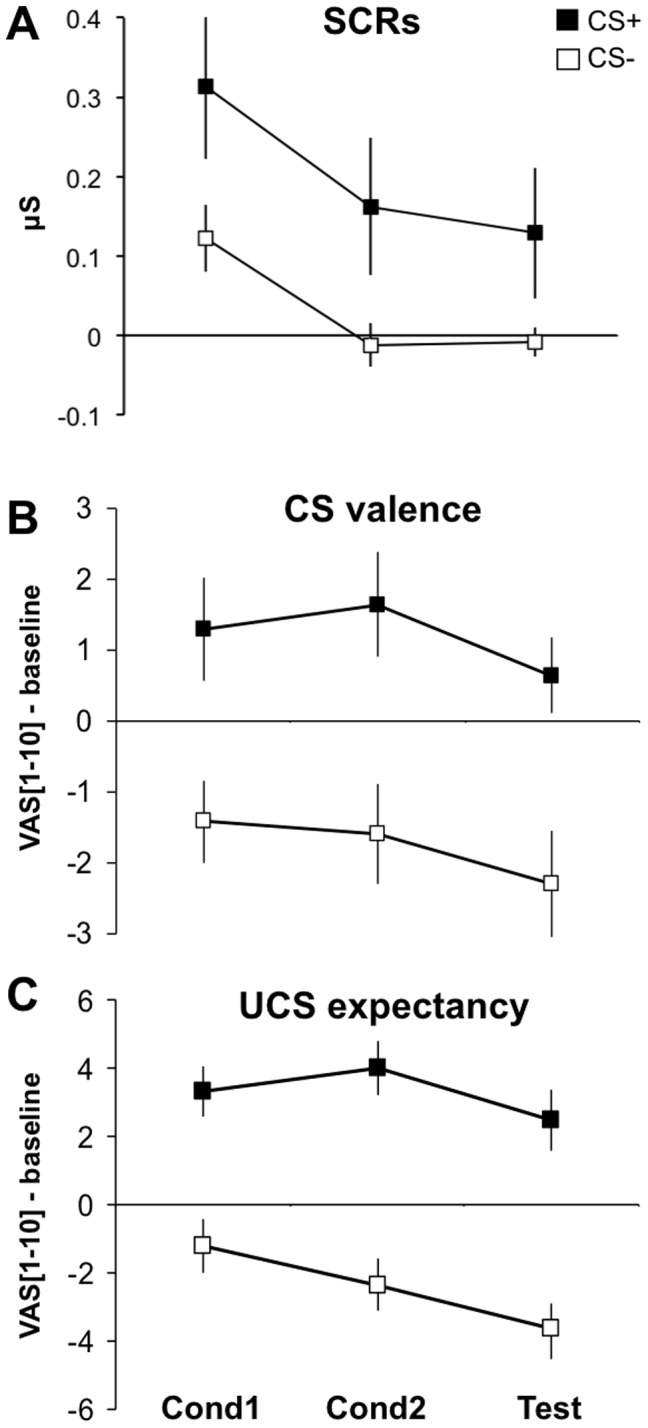
Uninstructed fear (study 2): Behavior. SCRs (**A**), CS valence (**B**), and UCS expectancy (**C**) during the two conditioning runs (UF-Cond1, UF-Cond2) and the test run (UF-Test). SCR analysis (**A**) was restricted to unpaired CS+s and CS−s, and responses were averaged across each run. Rating data (**B, C**) are normalized to the baseline rating given after habituation and before conditioning. Responses to the refresher CS at the outset of the UF-Test run (see **Methods**) are not shown. VAS, visual analog scale. Error bars: s.e.m.

### Unconditioned Stimulus

In both studies, unpleasant electrodermal stimulation was used as UCS. Stimuli were applied through Ag-AgCl electrodes fixed to the right wrist using a Digitimer DS7A stimulator (Digitimer, Welwyn Garden City, UK). Prior to scanning, the level of electrodermal stimulation to be received was determined via a standardized dial-up procedure in which stimuli were increased gradually to a level of intensity experienced as “uncomfortable but not painful”, with the aim of standardizing perceived UCS aversiveness across subjects [24].

**Figure 4:**
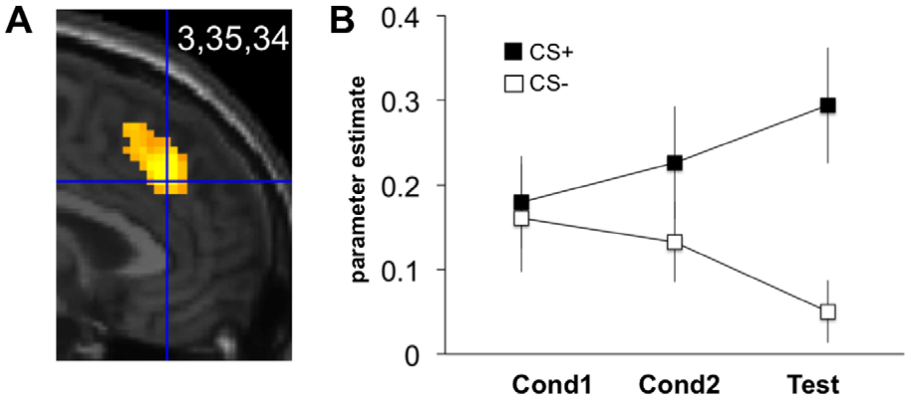
Uninstructed fear (study 2): Rostral dmPFC/dACC activation. (**A**) Contrast ‘unpaired CS+>CS−’ at testing (UF-Test run). Display threshold: p<0.001 uncorrected. Activations superimposed on a canonical structural image. (**B**) Parameter estimates from the peak voxel during all three runs (UF-Cond1, UF-Cond2, UF-Test). Error bars: s.e.m.

### Procedure

#### Instructed fear (study 1)

After the dial-up procedure and before the subsequent IF test inside the scanner, subjects were told that one of two CSs (yellow and blue square, physically shown to them once for the purpose of habituation) might be paired with a UCS during the test. For the CS+, participants were informed that the “stimulation could occur at any time while the corresponding color was presented”. The CS− represented the “safe” condition, indicating that “no shock would occur at any time”. Stimuli were counterbalanced across subjects. The scanning experiment consisted of two test runs (IF-Test 1, IF-Test 2) of about 5 min each, between which scanning was stopped. A run began with a rest period of 20 s after which each CS was presented five times in pseudo-random order. A CS lasted 12 s and was followed by an 18-s inter-trial interval (ITI) during which a fixation cross was presented. No UCS was given at any time. Subjects were debriefed after the scanning with special regard to their expectancy of a UCS.

**Table 1:** Areas activated during Instructed and Uninstructed Fear.

Study	Contrast	Region	p uncorr.	T-Value	Z-Value	x [mm]	y [mm]	z [mm]
IF-Test	CS+>CS−	dACC/dmPFC	<0.001	5.40	4.88	6	36	33
		dmPFC (left)	<0.001	3.80	3.60	−36	48	24
		dmPFC (right)	<0.001	4.52	4.19	42	45	18
		dorsal midbrain	<0.001	7.10	6.07	9	−15	−12
		insula cortex (anterior left)	<0.001	7.66	6.43	−27	24	−6
		insula cortex (anterior right)	<0.001	8.04	6.66	36	18	−9
		temporal cortex (mid right)	<0.001	4.81	4.43	51	−27	−9
		posterior cingulate cortex	<0.001	4.91	4.51	0	−18	24
		posterior parietal cortex (right)	<0.001	5.56	5.00	57	−45	48
		temporal cortex (superior left)	<0.001	4.29	4.00	−57	−39	18
	CS−>CS+	hippocampus (left)	<0.001	4.23	3.96	−30	−36	−6
		hippocampus (right)	<0.001	4.10	3.85	33	−36	−3
		insula cortex (posterior right)	<0.001	5.11	4.66	36	−15	18
		parahippocampus (right)	<0.001	4.82	4.43	33	−18	−21
		postcentral cortex (left)	<0.001	5.14	4.69	−48	−18	45
		SMA/primary motor cortex	<0.001	7.31	6.21	9	−21	66
		vmPFC	<0.001	5.71	5.12	−3	48	−18
UF-Test	CS+>CS−	dACC/dmPFC	<0.001	5.02	4.72	3	35	37
		insula cortex (anterior left)	<0.001	6.04	5.55	−30	23	−5
		insula cortex (anterior right)	<0.001	6.25	5.71	30	23	−8
		preSMA	<0.001	4.03	3.86	12	20	64
		supramarginal cortex (left)	<0.001	4.67	4.42	−60	−46	34
		supramarginal cortex (right)	<0.001	3.96	3.55	63	−46	28
		thalamus	<0.001	4.05	3.88	6	−22	1
	CS−>CS+	calcarine cortex (left)	<0.001	3.70	3.56	−12	−52	10
		calcarine cortex (right)	<0.001	4.05	3.88	12	−52	10
		dmPFC (left)	<0.001	3.99	3.83	−18	35	43
		dmPFC (right)	<0.001	4.63	4.38	24	29	43
		hippocampus (left)	<0.001	4.03	3.86	−24	−37	−14
		hippocampus (right)	<0.001	4.39	4.18	27	−19	−20
		insula cortex (posterior right)	<0.001	5.25	4.91	36	−10	16
		occipital cortex (mid right)	<0.001	4.66	4.41	45	−70	28
		occipital cortex (superior left)	<0.001	3.74	3.61	−27	−76	37
		occipital cortex (superior right)	<0.001	3.77	3.63	30	−79	40
		paracentral lobule (right)	<0.001	4.13	3.95	6	−34	61
		primary motor cortex (left)	<0.001	4.17	3.99	−54	−16	43
		primary motor cortex (right)	<0.001	5.05	4.74	51	−13	55
		rectus (left)	<0.001	4.58	4.34	−6	44	−20
		temporal cortex (mid left)	<0.001	3.80	3.66	−45	−7	−23
		temporal cortex (mid right)	<0.001	4.27	4.07	60	−7	−23
		temporal cortex (superior left)	<0.001	3.73	3.60	−54	−4	−11
		vmPFC	<0.001	4.58	4.34	−6	44	−20

Areas activated in the contrasts CS+>CS− and CS−>CS+ at p_uncorr_<0.001, k = 10, in Instructed Fear (study 1, IF-Test) and Uninstructed Fear (study 2, UF-Test).

#### Uninstructed fear (study 2)

Inside the scanner, subjects were shown once the two neutral visual stimuli (two Rorschach pictures [25]) later to become the CS+ and CS−, for the purpose of habituation. Subjects were told that the two stimuli would be presented in random order and that they might experience electrodermal stimulation. No instructions were given about stimulus contingencies, or time point or frequency of UCS delivery. Stimuli were counterbalanced across subjects. The experiment consisted of two conditioning runs (UF-Cond1, UF-Cond2) and one UF test run (UF-Test) of about 8 min each, between which scanning was stopped. In each of the two conditioning runs, subjects saw 12 CS+s, of which 6 were paired with a UCS (50% partial reinforcement), and 12 CS−s which were never paired with the UCS. The test run started with the presentation of one paired CS+ (“refresher” CS). It then continued with 12 unpaired CS+s and 12 CS−s which were used to assess fear appraisal/expression in the absence of further reinforcement (i.e., learning). CSs were presented for 5 s in a pseudo-randomized order and co-terminated with a UCS in the case of pairing. During the inter-trial interval (ITI) which varied between 13.5 and 16.5 s subjects saw a fixation cross. After habituation and before conditioning (baseline) and after each run, subjects rated their UCS expectancy and their perceived CS+ and CS− valences on a 10-point visual analog scale (expectancy: from ‘absolutely sure no shock will occur’ to ‘absolutely sure a shock will occur’; valence: from unpleasant to pleasant).

### Skin Conductance

SCRs were acquired using Ag-AgCl electrodes attached to the distal phalanges of the second and the third digits of the left hand and recorded continuously at a sampling rate of 5000 Hz using a BrainAmps ExG MR system (BrainProducts, Munich, Germany). Off-line analysis of SCR waveforms was performed using in-house software (Avg_q [26]). Data were filtered for (mainly scanner induced) high frequency artifacts with a 0.5 Hz low-pass filter. SCR quantification involved the following steps. First, the SCR waveform was baseline corrected by subtracting the average skin conductance level 2 seconds prior to stimulus onset (SCLcorr). Second, presence of an SCR was scored if a positive deflection was present that reached its half maximum in a 1.5 s to 2.5 s time window after stimulus onset. Third, the amplitude of thus defined SCRs was calculated as the mean SCLcorr during a 2 s window centered on the local maximum within a 3 s to 8 s time window after stimulus onset. For trials without valid SCRs, SCLcorr values where averaged from a 2 s time window centered on each individual’s average time to peak latency. For both studies the mean SCLcorr values were entered into separate repeated-measures analyses of variance (rm-ANOVA) with the factors Stimulus (study 1: CS− and CS+; study 2: CS− and unpaired CS+) and Time.

### Functional Imaging

Functional images were acquired on a Siemens 3T tim-TRIO magnetom (Erlangen, Germany) equipped with an 8-channel head coil. BOLD-sensitive functional volumes were recorded with an echo-planar T2*-weighted (EPI) sequence (study 1: TR = 2 s, TE = 30 ms, flip angle = 90°, FOV = 192×192 mm^2^, voxel size = 3*3*3 mm^3^, water suppression; study 2: TR = 2.5 s, TE = 30 ms, flip angle = 90°, FOV = 192×192 mm^2^, voxel size = 3*3*3 mm^3^, fat suppression). Directly after image acquisition, all EPI volumes were run through a rigid-body transformation to correct for head motion and through a distortion correction algorithm [27], both implemented at the MRI scanner. After the functional runs, a T1-weighted anatomical reference scan was recorded (TR = 2200 ms, TE = 4.11 ms, flip angle = 12°, FOV = 256×256 mm^2^, voxel size = 1*1*1 mm^3^).

Data preprocessing and statistical analysis were performed using SPM8 (Welcome Trust Centre of Imaging Neuroscience, London; for details, see [28]. After discarding the first 5 volumes of every run, the first remaining functional volume of the first run and the anatomical scan were manually rigid-body transformed to match the MNI (Montreal Neurological Institute) standard brain’s AC-PC orientation. Then, all functional volumes were realigned to the first volume of the first run to correct for head motion and spatially normalized into the MNI reference system. A subsequent spatial smoothing step with a three-dimensional isotropic Gaussian kernel (8 mm FWHM) was applied to increase signal-to-noise ratio and to compensate for inter-individual differences in location of corresponding functional areas. Signal time courses were high-pass filtered (128 s) to remove low-frequency noise.

At the single-subject level, for each study different multiple regression models (general linear model [GLM]) were fitted voxel-wise to voxel signal time courses. In study 1 (IF), the model contained one (unpaired) CS+ and one CS− regressor which were both constructed from 12-s “box cars” (on/off) at each stimulus onset, plus one constant for each run (IF-Test1 and 2) and a global constant. A second model analyzed CS+ and CS− responses in each run separately. In study 2 (UF), the model contained one paired CS+, one unpaired CS+, and one CS− regressor for each of the UF-Cond1 and 2 runs and one refresher CS+, one unpaired CS+, and one CS− regressor for the UF-Test run, which were all constructed from 5-s box cars. In addition, there were three constants for each of the runs and one global constant. CS regressors were convolved with a canonical hemodynamic response function. The resulting parameter estimate (“beta”) images for the unpaired CS+ and the CS− regressors were subjected to voxel-wise group-level random effects analyses separately for each study using SPM’s “full factorial” model with factors Stimulus (unpaired CS+, CS−) and, where applicable, Time (study 1, model 2: IF-Test1, IF-Test2; study 2: UF-Cond1, UF-Cond2, UF-Test). The model allows for correcting for a possible non-sphericity of the error term (here, dependence of factor levels). Unpaired CS+ vs. CS− contrasts were calculated using voxel-wise one-tailed t-tests. Parameter estimates of paired CS+ regressors did not enter voxel-wise group-level random effects analyses. Correction for multiple comparisons at an alpha threshold of p<0.05 was limited to a predefined rostral dmPFC/dACC ROI (“small volume correction”, SVC) and followed Gaussian random field theory (family-wise error rate (FWE) method). Additional exploratory analyses that did not serve to test our main hypothesis but were hypothesis-generating in nature used an uncorrected alpha threshold of p<0.001.

## Results

### Instructed Fear (Study 1)

#### Behavioral and physiological data

At debriefing after test, all subjects indicated that they had expected to receive electrodermal stimulation during presentation of the CS+, until some point in time when expectancy started to decrease. The latter suggests that, while certainly precluding fear learning by reinforcement, our measure of never delivering the announced UCSs during the IF test also had the side effect of eventually violating subjects’ threat expectations and thus to potentially induce another learning process that can initiate extinction [29]. Repeated-measures analysis of variance (rm-ANOVA) of SCR data showed a significantly higher response towards the CS+ compared to the CS− [main effect of Stimulus (CS+, CS−): F(1,16) = 13.44, p = 0.002] that habituated over time [main effect of Time (IF-Test1, IF-Test2): F(1,16) = 10.69, p = 0.005] but did not extinguish yet [Stimulus by Time interaction: F(1,16) = 0.1, p = 0.754] (**Figure 1**), i.e. across time, the signal decline over CS+ trials was not significantly different from that over CS− trials.

#### Imaging data

Our previous study on conscious threat appraisal had identified rostral dmPFC/dACC activation peaking at coordinates x,y,z = -8,38,28 [9]. Like in our subsequent catastrophizing study [12], we therefore defined our rostral dmPFC/dACC ROI as a box of dimensions x,y,z = 20,16,16 mm centered around 0,38,28 (box delineated in **Figures S2, S3 and S4**). Midline-centering (x = 0) served to assure equivalent bilateral mPFC coverage. As predicted, this yielded significant CS+>CS− activation differences at 6,36,33 (p<0.001 SVC; **Figure 2A**), further confirming the postulated role for the rostral dmPFC/dACC in IF [10]. The activation cluster spanned both the cingulate cortex and dorsally adjacent parts of the mPFC. Crucially, due to the nature of our IF paradigm, this activation cannot be explained by fear learning via Pavlovian conditioning. rm-ANOVA on parameter estimates in this peak voxel, extracted from a model that separated the two test runs (model 2, see **Methods**), showed a significant differential neural reaction (CS+>CS−) in this area [main effect of Stimulus (CS+, CS−): F(1,16) = 34.67, p<0.001] that neither habituated [main effect of Time (IF-Test1, IF-Test2): F(1,16) = 0.69, p = 0.42] nor extinguished [Stimulus by Time interaction: F(1,16) = 0.29, p = 0.601] (**Figure 2B**).

### Uninstructed Fear (Study 2)

#### Behavioral and physiological data

Rather than by instruction, a stimulus can also come to signal threat by experience. **Figure 3** suggests this was the case in study 2 where subjects were first fear-conditioned (runs UF-Cond1, UF-Cond2) and then tested for UF in the absence of further reinforcement (UF-Test). Separate rm-ANOVAs on SCRs, CS valence and UCS expectancy ratings, respectively, each with factors Stimulus (CS+, CS−) and Time (UF-Cond1, UF-Cond2, UF-Test), revealed a significantly higher reaction towards the CS+ as compared to the CS− [main effect of Stimulus: SCR: F(1,16) = 6.85, p = 0.019; valence: F(1,16) = 8.92, p = 0.009; expectancy: F(1,16) = 28.03, p<0.001]. The reaction habituated [main effect of Time: SCR: F(2,32) = 9.5, p = 0.003; valence: F(2,32) = 6.58, p = 0.008; expectancy: F(2,32) = 14.82, p<0.001] but was not detectably modulated by the omission of reinforcement in UF-Test [no significant Stimulus by Time interactions: SCR: F(2,32) = 0.34, p = 0.658; valence: F(2,32) = 0.47, p = 0.553; expectancy: F(2,32) = 3.36, p = 0.063]. These data thus allow us to classify the UF-Test run, where no further fear learning (conditioning) occurs, as a situation of fear appraisal/expression. Interestingly, while in study 1 subjects UCS expectations had apparently decreased to some extent by the end of the experiment (see above), the explicit expectancy ratings provided by the subjects in this study argue against relevant expectancy updating during the UF test. Notably, while relative expectancy ratings (CS+ minus CS−) increased from UF-Cond1 to UF-Cond2 [t(16) = 3.35, p = 0.004], there was no detectable change from UF-Cond2 to UF-Test [t(16) = 0.30, p = 0.765; both two-tailed paired t-tests] (compare also **Figure 3C**).

#### Imaging data

To test whether UF also activates the rostral dmPFC/dACC, we searched for activation during UF-Test in the same ROI as used in study 1 (centered at 0,38,28). As predicted, this yielded significant CS+>CS− activation differences in a very similar location, again spanning cingulate and medial prefrontal cortices (3,35,34; z score = 4.20, p<0.001 SVC; **Figure 4A**). The peak-voxel parameter estimates from all three experimental phases in **Figure 4B** suggest a neural reaction towards the CS+ that gradually developed during conditioning (runs UF-Cond1, UF-Cond2) but only fully expressed during testing (run UF-Test). This effect persisted, when regarding the mean parameter estimates across all voxels in the aforementioned ROI (**Figure S1)**. Rm-ANOVA on the peak-voxel estimates yielded a Stimulus by Time interaction [F(2,32) = 3.92, p = 0.036; main effect of Stimulus: F(1,16) = 10.68 p = 0.005; main effect of Time: F(2,32) = 0.04, p = 0.965]. Further confirming the visual impression, planned post-hoc one-tailed t-tests on CS+>CS− difference scores showed no significant activation during early conditioning [UF-Cond1: t(16) = 0.28, p = 0.393], an already significant, though rather weak, reaction during late conditioning [UF-Cond2: t(16) = 2.11, p = 0.025] and a strong and highly significant reaction to the CS+ during testing [UF-Test: t(16) = 3.81, p = 0.001]. The reaction in UF-Test was larger than in UF-Cond1 [t(16) = 2.59, p = 0.02] and in UF-Cond2 [t(16) = 2.23, p = 0.041; both two-tailed paired t-tests]. Of note, this stands in contrast to the absence of a Stimulus by Time interaction in the SCR data above where the CS+>CS− effect was significant from the first time point on. Hence, the neural effect followed rather than preceded the physiological effect.

### Other Regions

Exploratory whole-brain analysis at an uncorrected threshold of p<0.001 (see Methods) also suggested activation of more posterior dmPFC/dACC areas in both study 1 (IF) and study 2 (UF) (compare extended activation clusters in **Figures 2A** and **4A** **;** **Table 1** gives a full list of activations). In line with the proposed functional segregation between more posterior and rostral dmPFC/dACC [5], posterior dmPFC/dACC activation in study 2 showed a different temporal profile across the three runs from rostral dmPFC/dACC activation, being most pronounced during late conditioning (UF-Cond1, 2; **Figure S2**). Interestingly, the vmPFC, which has been reported to be deactivated during conditioning [30] also seemed to be markedly deactivated during both IF and UF testing (study 1 (IF): -3,48,-18; z score = 5.12; study 2 (UF): -6,44,-20; z score = 4.34; **Figure S3 and S4**). We nevertheless stress the descriptive nature of these results which we will not discuss further.

## Discussion

Our findings from two independently tested fear expression paradigms considerably advance our understanding of medial prefrontal function in fear/anxiety: they strongly indicate that a previously described threat-responsive rostral dmPFC/dACC area makes at most a minor contribution to fear learning while at the same time they confirm the threat-responsiveness of this region. That is, the area was primarily active during a situation of pure fear appraisal/expression in the absence of fear learning (UF-Test) but tended to start responding already during the course of prior conditioning. In addition, the observation from the uninstructed fear expression paradigm (UF, study 2) that the area only started to respond after peripheral-physiological conditioned responding (SCRs) had already been registered further replicates previous results that the area is not directly involved in physiological fear expression. In combination with the data discussed below, this supports our hypothesis that one of the major functions of the rostral dmPFC/dACC is the appraisal of threat.

Threat situations induce a host of processes including attentional deployment, appraisals of the threat content of the situation, and subsequent autonomic, hormonal, motor and subjective-experiential threat reactions. Threat reactions change the external and internal environment and can therefore become emotional stimuli in their own right, inducing a new cycle of attending, appraising and reacting. Finally, the described emotion generation processes are often intermingled with associative learning and recall of threat contingencies. This complexity of the organism’s threat response is a challenge for any functional-neuroanatomical examination. Nevertheless, the current state of research permits some relatively safe conclusions with regards to rostral dmPFC/dACC function in threat. First, the present data and two aforementioned studies [9], [12] conclusively show that neural activity in the rostral dmPFC/dACC is dissociated from responding at the peripheral-physiological level, making it unlikely that the area is directly engaged in the expression of physiological fear responses. As argued earlier, better candidates for this function can be found in more posterior parts of the dmPFC/dACC [13]–[17] (reviewed in [5]), or the insular cortex [31]. Second, the area is particularly active when threat is processed consciously or explicitly [9], [12], a claim that was not tested in the present study but that resonates with evidence from studies outside the domain of fear where the rostral dmPFC is active when emotional stimuli are evaluated explicitly (see [32] for meta-analysis). Third, the area is down-regulated when threat is reappraised in a less negative fashion [8] and hyperactive in subjects that catastrophize [12], suggesting the area is particularly concerned with the negative aspects of a threat situation. In sum, these data suggests a conscious negative threat appraisal function for the rostral dmPFC/dACC. However, it would be premature to conclude that the area is exclusively concerned with valence-specific negative threat appraisal but it may have a more general function in conscious emotional evaluation irrespective of stimulus valence [33]. A possible alternative explanation that the area supports fear acquisition has been made unlikely by our present findings. Yet another possible alternative explanation for rostral dmPFC/dACC activation during threat is that it mediates the subjective-experiential, or feeling, aspect of fear. Arguing against this explanation is the observation that subjects still report high levels of subjective anxiety even if rostral dmPFC/dACC activation is entirely abolished [9]. We emphasize however that the latter result was obtained on the basis of post-hoc anxiety ratings and should therefore be tested again in an optimized paradigm.

One question mark that the present results raise is why subjects in study 2 (UF) explicitly evaluated CSs as predicting a UCS and being of negative valence already during the conditioning runs, but only strongly activated their rostral dmPFC/dACC later, during testing. If the rostral dmPFC/dACC is responsible for explicit threat appraisals, its activation should parallel those. One possibility might be that the area is less interested in the threatening properties of external stimuli or their contingencies but in the internal consequences of a threat situation. It might thus monitor and judge changes in attention, bodily states, or feelings that occur during threat. In catastrophizers, the negative interpretation of such internal changes as signals of impending harm can cause a state of “fear of fear” that may contribute to the development of pathological anxiety [34], [35]. Such an interpretation of our findings would tie in with the observation that normal subjects who are genetically pre-disposed to develop panic disorder show a hyper-activation of the rostral dmPFC/dACC during Pavlovian fear conditioning that correlates with a subjective over-estimation of their conditioned fear reactions [12]. The interpretation is also supported by a very recent study showing rostral dmPFC/dACC activity during instructed fear of an interoceptive threat (a breathing challenge) that was correlated with a trait measure of fear of somatic symptoms [36].

The present design where reinforcement was deliberately omitted during fear testing has the unavoidable disadvantage that we cannot definitively exclude extinction learning as an alternative explanation for dmPFC/dACC activation at test. Extinction is thought to result from the prediction error that is registered when an expected aversive reinforcement does not occur [29]. In study 1 (IF), we tried to prevent such expectation violation by only instructing subjects that the UCS “might” occur. Nevertheless, subjects’ post-experimental self-report suggest they did update their UCS expectancies to a certain degree, even though this did not express in a concomitant reduction of fear responding (SCRs). Study 2 (UF) contained two elements that we hoped would slow down extinction during testing: i) a low reinforcement ratio of 50% during conditioning and ii) the presentation of a single paired “refresher” CS+ at the outset of the test run that, together, should relatively reduce prediction errors when the UCS is omitted at test. To better assess potential expectancy changes, we further asked subjects to provide quantitative expectancy ratings before and after every run. In contrast to study 1, there was no evidence for any expectancy updating. Furthermore, as in study 1, there was no evidence for actual extinction of conditioned skin conductance responding. Both would speak against the occurrence of prediction errors. Nevertheless, it is theoretically possible that subjects only change their expectations (and consequentially their conditioned responding) after having sampled a sufficient amount of prediction errors. The question whether dmPFC/dACC activation might reflect a prediction error-type mechanism rather than threat appraisal can thus not be conclusively answered from our data. However, the presence of rostral dmPFC/dACC activation in IF paradigms where reinforcement does occur at a rate corresponding to the instruction [8], [9], [19], [20] as well as the consistent observation that extinction is spared after dorsal mPFC lesions in rodents [37]–[40] would suggest the question should be answered in the negative. Further research will be required to clarify this issue. A final limitation of our study that needs to be mentioned is that all findings reported here emanate from analyses that were secondary to the original purposes of the two studies.

To conclude, we have presented and discussed convergent evidence that speaks for an involvement of the rostral dmPFC/dACC in conscious negative threat appraisal. Cognitive psychotherapy tries to heal pathological anxiety by making patients aware of the unrealistic nature of such appraisals and teaching them to replace their negative thoughts by a more positive interpretation of the feared situation. It is an intriguing speculation that the rostral dmPFC/dACC might be at the source of negative thinking in pathological anxiety and that therapeutic progress might express in a silencing of rostral dmPFC/dACC activation. It would also be interesting to investigate whether rostral dmPFC/dACC inhibition, perhaps possible with tools like transcranial direct current stimulation, can alleviate negative cognitions and accompanying anxiety. In turn, in a safe therapy setting a patient might benefit from dmPFC/dACC stimulation when being guided to re-appraise fear or anxiety inducing situations or memories. A recent finding that threat enhances rostral dmPFC/dACC coupling with the amygdala [41] suggests the area could be a promising entry point into the fear system. The present line of research thus opens up a potentially promising avenue for translational research.

## Supporting Information

Figure S1 **Uninstructed fear (study 2): Rostral dmPFC/dACC activation.** Mean parameter estimates across all voxels in a predefined rostral dmPFC/dACC ROI as a box of dimensions x,y,z = 20,16,16 mm centered around 0,38,28 during all three runs (UF-Cond1, UF-Cond2, UF-Test). Error bars: s.e.m.
(TIFF)

Figure S2 **Uninstructed fear (study 2): posterior dACC activation.** (**A**) Contrast ‘unpaired CS+>CS−’ at late conditioning (UF-Cond2 run). Display threshold: p<0.001 uncorrected. Activations superimposed on a canonical structural image with the rostral dmPFC/dACC ROI depicted as a square of lighter grey. (**B**) Parameter estimates from the peak voxel during all three runs (UF-Cond1, UF-Cond2, UF-Test). Error bars: s.e.m.
(TIFF)

Figure S3 **Instructed fear (study 1): vmPFC activation.** (**A**) Contrast ‘CS−>CS+‘ across both test runs (IF-Test1, IF-Test2) (model 1, see **Methods**). Display threshold: p<0.001 uncorrected. Activations superimposed on a canonical structural image with the rostral dmPFC/dACC ROI depicted as a square of lighter grey. (**B**) Parameter estimates from the peak voxel, estimated separately for each test run (model 2). Error bars: s.e.m.
(TIFF)

Figure S4 **Uninstructed fear (study 2): vmPFC activation.** (**A**) Contrast ‘unpaired CS−>CS+‘ at testing (UF-Test run). Display threshold: p<0.001 uncorrected. Activations superimposed on a canonical structural image with the rostral dmPFC/dACC ROI depicted as a square of lighter grey. (**B**) Parameter estimates from the peak voxel during all three runs (UF-Cond1, UF-Cond2, UF-Test). Error bars: s.e.m.
(TIFF)
