# Supplementary figures and images for: Clarifying the Role of the Rostral dmPFC/dACC in Fear/Anxiety: Learning, Appraisal or Expression?
Source: PLoS One. 2012 Nov 26;7(11):e50120. doi: 10.1371/journal.pone.0050120 (PMC3506550; doi:10.1371/journal.pone.0050120)

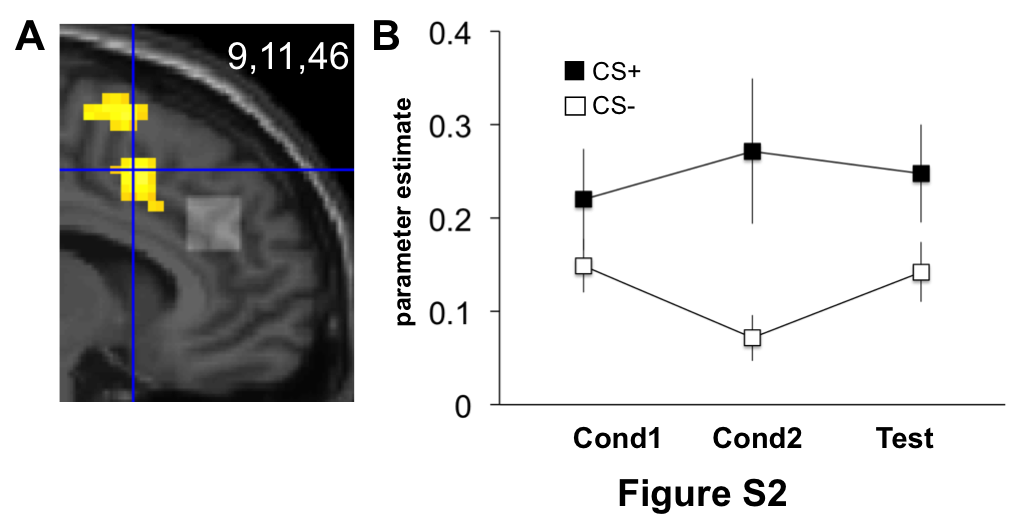

Supplement: Figure S2 — Uninstructed fear (study 2): posterior dACC activation. (A) Contrast ‘unpaired CS+>CS−’ at late conditioning (UF-Cond2 run). Display threshold: p<0.001 uncorrected. Activations superimposed on a canonical structural image with the rostral dmPFC/dACC ROI depicted as a square of lighter grey. (B) Parameter estimates from the peak voxel during all three runs (UF-Cond1, UF-Cond2, UF-Test). Error bars: s.e.m. (TIFF) [file pone.0050120.s002.tiff]

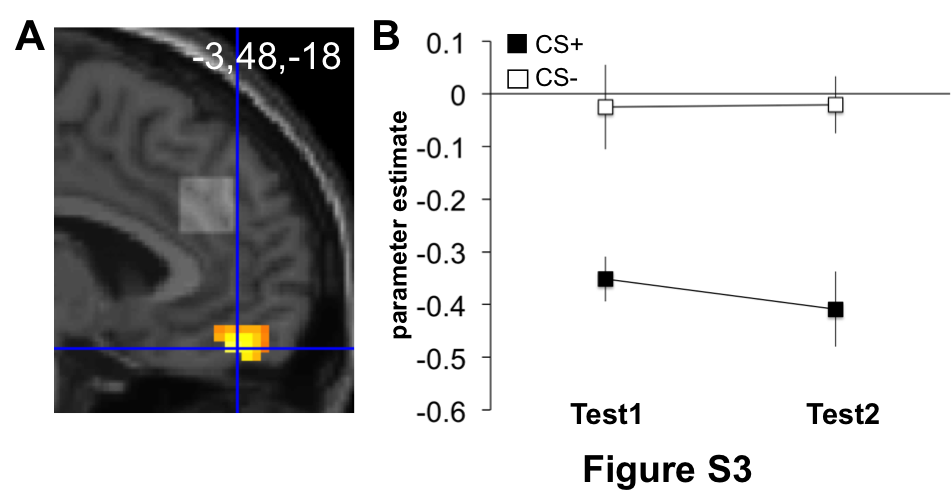

Supplement: Figure S3 — Instructed fear (study 1): vmPFC activation. (A) Contrast ‘CS−>CS+‘ across both test runs (IF-Test1, IF-Test2) (model 1, see Methods). Display threshold: p<0.001 uncorrected. Activations superimposed on a canonical structural image with the rostral dmPFC/dACC ROI depicted as a square of lighter grey. (B) Parameter estimates from the peak voxel, estimated separately for each test run (model 2). Error bars: s.e.m. (TIFF) [file pone.0050120.s003.tiff]

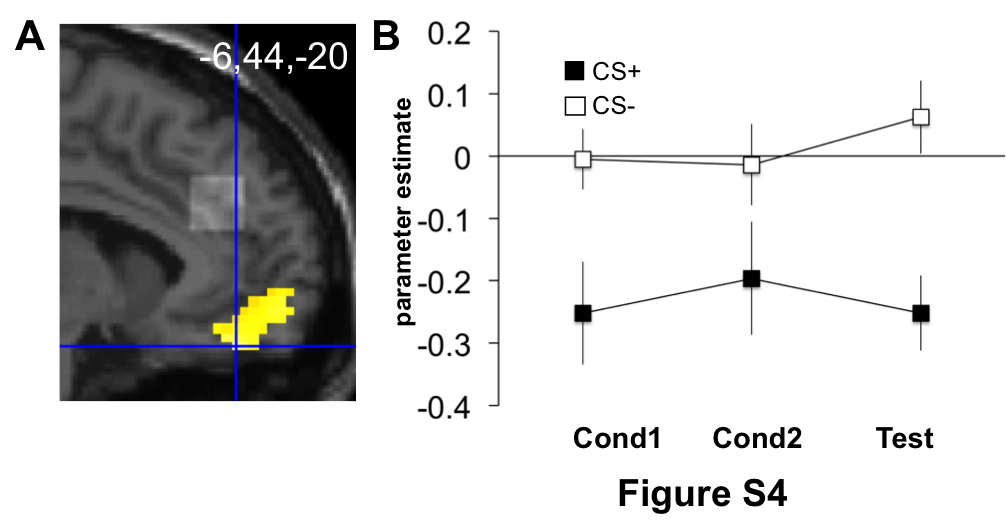

Supplement: Figure S4 — Uninstructed fear (study 2): vmPFC activation. (A) Contrast ‘unpaired CS−>CS+‘ at testing (UF-Test run). Display threshold: p<0.001 uncorrected. Activations superimposed on a canonical structural image with the rostral dmPFC/dACC ROI depicted as a square of lighter grey. (B) Parameter estimates from the peak voxel during all three runs (UF-Cond1, UF-Cond2, UF-Test). Error bars: s.e.m. (TIFF) [file pone.0050120.s004.tiff]
